# Non-invasive Vagus Nerve Stimulation (nVNS) as mini-prophylaxis for menstrual/menstrually related migraine: an open-label study

**DOI:** 10.1186/s10194-016-0684-z

**Published:** 2016-10-03

**Authors:** Licia Grazzi, Gabriella Egeo, Anne H. Calhoun, Candace K. McClure, Eric Liebler, Piero Barbanti

**Affiliations:** 1 Headache Center, Carlo Besta Neurological Institute and Foundation, Via Celoria 11, 20133 Milan, Italy; 2 Headache and Pain Unit, Istituto di Ricovero e Cura a Carattere Scientifico, San Raffaele Pisana, Via della Pisana 235, 00163 Rome, Italy; 3 Carolina Headache Institute, 6114 Fayetteville Rd., Suite 109, 27713 Durham, NC USA; 4 North American Science Associates Inc., 4050 Olson Memorial Highway, Suite 450, 55422 Minneapolis, MN USA; 5 electroCore, LLC, 150 Allen Road, Suite 201, 07920 Basking Ridge, NJ USA

**Keywords:** Menstrual migraine, Menstrually related migraine, Prophylactic treatment, Vagus nerve

## Abstract

**Background:**

Menstrual migraine and menstrually related migraine attacks are typically longer, more disabling, and less responsive to medications than non-menstrual attacks. The aim of this study was to evaluate the efficacy, safety, and tolerability of non-invasive vagus nerve stimulation for the prophylactic treatment of menstrual migraine/menstrually related migraine.

**Methods:**

Fifty-six enrolled subjects (menstrual migraine, 9 %; menstrually related migraine, 91 %), 33 (59 %) of whom were receiving other prophylactic therapies, entered a 12-week baseline period. Fifty-one subjects subsequently entered a 12-week treatment period to receive open-label prophylactic non-invasive vagus nerve stimulation adjunctively (31/51; 61 %) or as monotherapy (20/51; 39 %) on day −3 before estimated onset of menses through day +3 after the end of menses.

**Results:**

The number of menstrual migraine/menstrually related migraine days per month was significantly reduced from baseline (mean ± standard error, 7.2 ± 0.7 days) to the end of treatment (mean ± standard error, 4.7 ± 0.5 days; *P* < 0.001) (primary end point). Of all subjects, 39 % (95 % confidence interval: 26 %, 54 %) (20/51) had a ≥ 50 % reduction (secondary end point). For the other secondary end points, clinically meaningful reductions in analgesic use (mean change ± standard error, −3.3 ± 0.6 times per month; *P* < 0.001), 6-item Headache Impact Test score (mean change ± standard error, −3.1 ± 0.7; *P* < 0.001), and Migraine Disability Assessment score (mean change ± standard error, −11.9 ± 3.4; *P* < 0.001) were observed, along with a modest reduction in pain intensity (mean change ± standard error, −0.5 ± 0.2; *P* = 0.002). There were no safety/tolerability concerns.

**Conclusions:**

These findings suggest that non-invasive vagus nerve stimulation is an effective treatment that reduces the number of menstrual migraine/menstrually related migraine days and analgesic use without safety/tolerability concerns in subjects with menstrual migraine/menstrually related migraine. Randomised controlled studies are warranted.

## Background

Menstrual migraine (MM) without aura is defined as the *exclusive* occurrence of attacks on days −2 to +3 of menstruation in at least 2 of 3 consecutive menstrual cycles according to the *International Classification of Headache Disorders, 3rd edition (beta version) (ICHD-III beta)* appendix (i.e., requires further validation), and menstrually related migraine (MRM) without aura is *also* characterized by the occurrence of attacks on other days of the cycle [1]. More than 90 % of women with migraine attacks during menstruation have MRM [2]; the estimated prevalence among migraineurs has varied from 0.85 to 14.1 % for MM and from 3 to 71.4 % for MRM [3]. These conditions are believed to be a result of fluctuating oestrogen levels; steady or elevating levels are associated with a protective effect, whereas abrupt oestrogen withdrawals are associated with precipitation of migraine attacks [2, 4]. In the late luteal phase of the menstrual cycle, decreased oestrogen levels have been observed, which lead to serotonin declines and are likely responsible for the triggering of MM/MRM attacks just prior to menses [2, 4].

No acute or prophylactic therapies are currently approved specifically for the treatment of MM/MRM in the European Union or the United States [5, 6]. Acute treatments used for these conditions are the same as those used for non-menstrual migraine and include triptans, non-steroidal anti-inflammatory drugs (NSAIDs), and ergot derivatives [2, 6]. Prophylaxis comprises short-term and continuous treatments [2]. Short-term prophylactic therapies are administered only during the time of need (e.g., perimenstrually) and include triptans, oestrogen, and naproxen, whereas continuous prophylactic options such as hormonal contraceptives provide ongoing exposure to the treatment [2]. A systematic review of MM/MRM clinical trials indicated that evidence supporting most categories of prophylactic MM/MRM treatments is generally weak [6]. Clinical studies of triptans represent the strongest evidence to date for acute and preventive MM/MRM treatment, which supports almotriptan, naratriptan, sumatriptan, and zolmitriptan as acute therapies and frovatriptan, naratriptan, and zolmitriptan as preventive therapies [6].

Despite the general safety and tolerability of triptans with appropriate patient selection [2, 7, 8], the MM/MRM population may have unique challenges related to the adverse events (AEs) associated with these treatments. Compared with non-menstrual migraine attacks, MM/MRM attacks are generally longer lasting, more debilitating, more prone to recurrence, and less responsive to therapies such as triptans [9, 10]. Results from a large study showed that 44 % and 48 % of migraineurs were dissatisfied with triptan-associated tolerability and general/work-related functional ability, respectively [7], and these concerns may be even more prominent in the relatively treatment-refractory MM/MRM population [10]. Menses is generally considered to be a period of inherent discomfort [11], and the repeated treatments required to mitigate the effects of MM/MRM could further exacerbate the level of discomfort while providing only minimal response [10, 12]. Evidence-based guidelines for migraine suggest limiting the use of triptans to 2 headache days per week to reduce the risk of rebound or medication-overuse headache [8], and frequent use of these agents may lead to misuse/overuse and has been significantly associated with the development of chronic migraine [13, 14]. Thus, the prophylactic administration of triptans over the course of several days during menstruation coupled with the potential need for acute triptan therapy in women with MM/MRM could complicate the condition [12, 13]. Patients may be unwilling to accept the AE burden and/or potential complications of adhering to monthly prophylactic MM/MRM therapies [15], defining a need for alternative treatment options among this population.

Non-invasive vagus nerve stimulation (nVNS) (gammaCore®; electroCore, LLC; Basking Ridge, NJ, USA) provides neuromodulation that transfers electrical impulses transcutaneously to the cervical branch of the vagus nerve. In 4 open-label ≤ 12-week studies, nVNS demonstrated efficacy, safety, and tolerability as an acute/prophylactic therapy for migraine and chronic cluster headache [16–19]. Based on the treatment benefits observed in previous studies and the potential for reducing medication overuse and medication-associated AEs [16], we evaluated nVNS used as mini-prophylaxis for MM/MRM in this 24-week study of 56 subjects.

## Methods

### Study design

This investigator-initiated, multicentre, single-arm, open-label study of the efficacy, safety, and tolerability of prophylactic nVNS treatment for MM/MRM was conducted from January 2015 to October 2015. After providing informed consent for trial participation and publication of patient data, subjects entered a 12-week run-in (baseline) period of observation followed by a 12-week nVNS treatment period to receive open-label, short-term MM/MRM prophylaxis. Investigators obtained approval from the Ethical Committees of the Istituto di Ricovero e Cura a Carattere Scientifico (IRCCS), San Raffaele Pisana (identifier: SR_MM12/2014). All source documents and files are stored in the clinical trial centres.

### Subjects

All subjects were enrolled from two Italian sites and were required to have a regular menstrual cycle. Inclusion criteria specified that subjects were to be 18 to 50 years of age. Subjects had a > 1-year history of migraine with or without aura and a diagnosis of MM/MRM without aura according to *ICHD-III beta* criteria [1]. Key exclusion criteria at the time of enrolment were another diagnosis of a primary headache disorder, such as chronic migraine, secondary headache disorder, or other neurological or severe systemic disease; current or previous vagal disturbances; a change in prophylactic medication type or dosage within 1 month before enrolment; previous failure of ≥ 3 prophylactic treatment classes; and participation in another clinical trial.

### Intervention

**Fig. 1:**
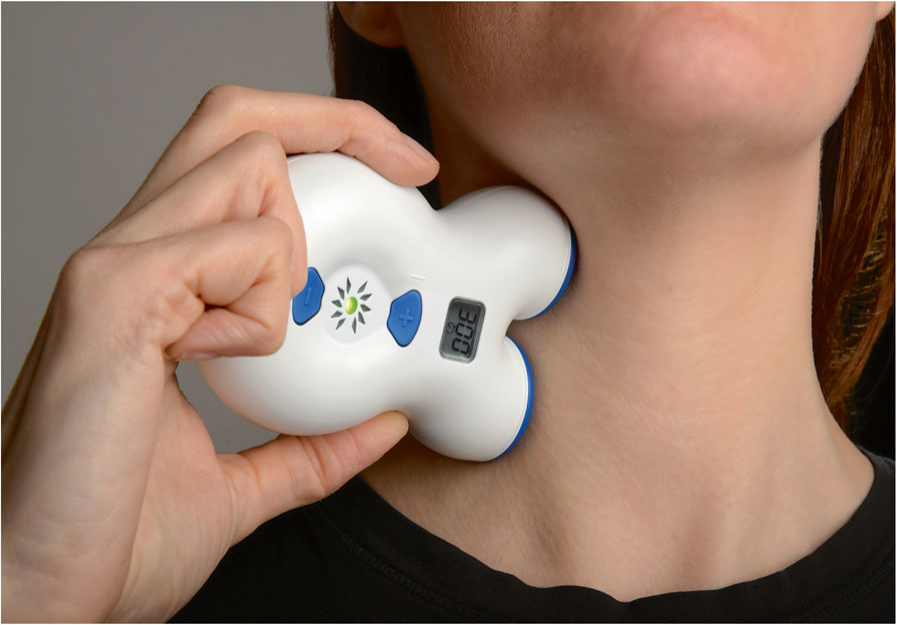
Non-invasive Vagus Nerve Stimulation Device. Image provided courtesy of electroCore, LLC

The nVNS devices (Fig. 1) for this study, supplied by electroCore, LLC, produce a proprietary low-voltage electrical signal comprising a 5-kHz sine wave burst lasting for 1 millisecond (five sine waves, each lasting 200 microseconds), with such bursts repeated once every 40 milliseconds (25 Hz), generating a 24-V peak voltage and 60-mA peak output current. Subjects received comprehensive training on appropriate device placement and adjustment of stimulation intensity for optimal dose administration. Bilateral 2-min stimulations were administered to the cervical branch of the vagus nerve 3 times daily at 8 am, 1 pm, and 8 pm (i.e., 6 stimulations total per day) after application of conductive gel to the 2 stainless steel contact surfaces. This procedure was performed from −3 days before estimated onset of menstruation through +3 days after the end of menstruation during each cycle of the 12-week nVNS treatment period. Treatment duration per month in this study was 10 to 14 days based on the actual days of menstruation. Doses of any prophylactic medications were kept stable throughout the study. Subjects were allowed to use their usual acute analgesics (alone or in combination), which included triptans, NSAIDs, and a butalbital/caffeine/propyphenazone combination medication.

### Study end points

The primary end point was the mean change in number of MM/MRM days per month between the baseline and nVNS treatment periods. Secondary end points included the proportion of subjects with a ≥ 50 % reduction in the mean number of MM/MRM days from the baseline period to the nVNS treatment period and mean changes between the two study periods in analgesic use, migraine disability, pain intensity, and allodynia. Exploratory analyses of proportions of subjects who achieved various levels of improvement in migraine disability were also performed.

### Data collection

At baseline, subjects underwent a physical and neurological examination and a live interview administered via a detailed, semistructured questionnaire on their 1) clinical migraine features; 2) demographics, lifestyle, and behavioural factors; and 3) comorbidities and concomitant medications [20]. During the study periods, subjects used diary cards to record their MM/MRM days and analgesic use on a daily basis. This information was used to calculate monthly averages for each 3-month study period. Data on migraine disability were collected using the 6-item Headache Impact Test (HIT-6™) [21] based on the last 4 weeks of each study period and the MIDAS based on the entire 3 months of each study period [22]. Scores on the HIT-6 range from 36 to 78 points, MIDAS scores range from 0 to 21+ points, and MIDAS grades range from 1 to 4, with higher values indicating greater disability for each of these assessments [21, 22]. Pain intensity for each migraine attack was rated using question B of the Migraine Disability Assessment (MIDAS) on a scale from 0 to 10, with higher values indicating greater intensity, and was averaged at the end of each 3-month study period [22]. Data on allodynia were collected using the 12-item Allodynia Symptom Checklist (ASC-12), with scores ranging from 0 to 24 points and higher scores indicating greater severity [23]. The investigator collected safety and tolerability data by asking participants to provide subjective responses at each study visit regarding any AEs after nVNS use.

### Statistical analyses

Subjects who received at least 1 nVNS treatment were included in the intent-to-treat (ITT) population for efficacy analyses. Mean ± standard error of the mean (SEM) changes between the two study periods were calculated using data from week 12 of the nVNS treatment period minus data from the baseline visit, with negative values indicating a decrease over time. Two-sided statistical analyses were conducted using a significance level of *P* < 0.05; *P* values were derived from the *t* test. The exact Clopper-Pearson method was used to calculate the 95 % confidence interval (CI) for the proportion of subjects with a ≥ 50 % reduction in mean number of MM/MRM days between the two study periods. Missing data were imputed using the last observation carried forward method. Data for subjects who discontinued during the nVNS treatment period were imputed to baseline values and were included in all efficacy analyses, whereas subjects with missing data for both study periods were not included in the analyses. All statistical analyses were performed independently by North American Science Associates Inc. (Minneapolis, MN, USA) using SAS® 9.3 (SAS Institute Inc., Cary, NC, USA).

## Results

### Subjects

Of the 56 subjects screened, all were enrolled and entered the baseline period, including 6 subjects who were older than 50 years of age but met all other entry criteria. All subjects were diagnosed with MM/MRM without aura and had episodic migraine at enrolment. Demographic and baseline characteristics (Table 1) showed a mean age of 40.2 years. Most subjects had MRM (91 %), and the remaining 9 % had MM. For 7 subjects, the average number of headache days per month in the baseline period had increased to a range of 15 to 20 days; this increase was not sustained for more than 3 months, thereby excluding a diagnosis of chronic migraine. Five subjects discontinued from the study before entering the nVNS treatment period, including 2 subjects who became pregnant, 1 subject who entered menopause, 1 subject who underwent surgery, and 1 subject for whom no reason for study withdrawal was provided. The remaining 51 subjects met criteria for the ITT population. Of these, 5 subjects discontinued from the nVNS treatment period because of lack of efficacy, and 1 subject discontinued because of non–device-related AEs.

**Table 1:** Demographic and Baseline Characteristics^a^

Characteristic	Total Population (*n* = 56)
Age (y), mean ± SEM	40.2 ± 1.0
Age of onset (y), mean ± SEM	17.9 ± 1.3
Currently employed, No. (%)	49 (88)
Migraine type, No. (%)	
MRM	51 (91)
MM	5 (9)
Pain location, No. (%)	
Bilateral	17 (30)
Unilateral	39 (70)
Average duration of untreated/unsuccessfully treated attacks, No. (%)	
< 12 h	2 (4)
12 to < 24 h	15 (27)
24 to < 48 h	7 (13)
48 to < 72 h	14 (25)
≥ 72 h	18 (32)
Analgesic medications used, No. (%)	
NSAID	17 (30)
NSAID plus triptan	20 (36)
NSAID plus a butalbital/caffeine/propyphenazone combination medication	1 (2)
Triptan	18 (32)
Other prophylactic medication use, No. (%)^b^	33 (59)

Abbreviations: *MM*, menstrual migraine, *MRM* menstrually related migraine, *NSAID* nonsteroidal anti-inflammatory drug, *nVNS* non-invasive vagus nerve stimulation, *SEM* standard error of the mean

^a^Data represent values from the baseline period

^b^Amitriptyline (5/56), coenzyme Q10/ginkgolide B/riboflavin (tablets) plus magnesium (4/56), coenzyme Q10/ginkgolide B/riboflavin/magnesium (granulated) (1/56), coenzyme Q10/ginkgolide B/riboflavin/magnesium (granulated) plus amitriptyline (1/56), magnesium (3/56), β-blocker (2/56), calcium channel blocker (2/56), paroxetine (2/56), riboflavin (2/56), amitriptyline plus benzodiazepine (1/56), amitriptyline plus magnesium (1/56), β-blocker plus amitriptyline (1/56), botulinum toxin A (1/56), sodium valproate plus venlafaxine (1/56), sodium valproate plus paroxetine (1/56), tanacetum parthenium (1/56), sertraline (1/56), topiramate plus sertraline (1/56), topiramate plus gabapentin (1/56)

### Menstrual/Menstrually related migraine days

**Fig. 2:**
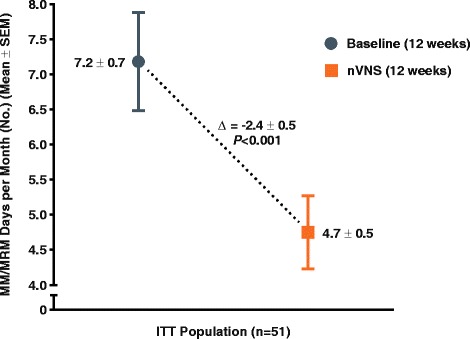
Change in Number of MM/MRM Days per Month (ITT Population)^a.^ Abbreviations: ITT, intention-to-treat; nVNS, non-invasive vagus nerve stimulation; SEM, standard error of the mean. ^a^Data are mean ± SEM; mean differences between treatment groups may not reflect calculated differences because of rounding; *P* values are from the *t* test; missing data were imputed using the last observation carried forward

Therapy with nVNS significantly reduced the mean number of MM/MRM days (primary end point) from baseline (7.2 ± 0.7 days) to the end of the treatment period (4.7 ± 0.5 days; *P* < 0.001) (Fig. 2). During the nVNS treatment period, 39 % (95 % CI: 26 %, 54 %) of all subjects (20/51) had a ≥ 50 % reduction in the mean number of MM/MRM days from baseline.

### Analgesic use

**Fig. 3:**
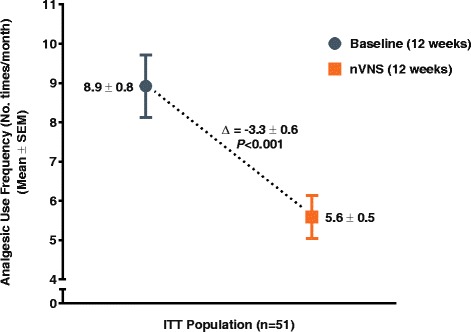
Change in Analgesic Use per Month (ITT Population)^a.^ Abbreviations: ITT, intention-to-treat; nVNS, non-invasive vagus nerve stimulation; SEM, standard error of the mean. ^a^Data are mean ± SEM; mean differences between treatment groups may not reflect calculated differences because of rounding; *P* values are from the *t* test; missing data were imputed using the last observation carried forward

Mean analgesic use was significantly reduced from 8.9 ± 0.8 times per month in the baseline period to 5.6 ± 0.5 times per month in the nVNS treatment period (*P* < 0.001) (Fig. 3).

### Migraine disability

**Fig. 4:**
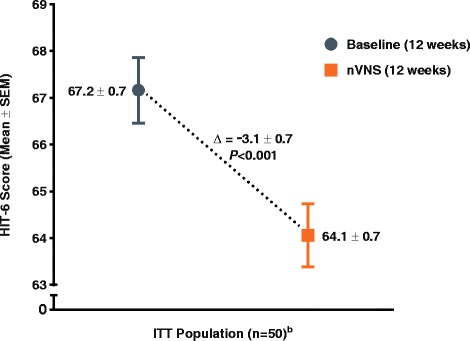
Change in HIT-6™ Migraine Disability (ITT Population)^a.^ Abbreviations: HIT-6, 6-item Headache Impact Test; ITT, intention-to-treat; nVNS, non-invasive vagus nerve stimulation; SEM, standard error of the mean. ^a^Data are mean ± SEM; mean differences between treatment groups may not reflect calculated differences because of rounding; *P* values are from the *t* test; missing data were imputed using the last observation carried forward. ^b^One subject had missing HIT-6 data for both the baseline and nVNS periods

A reduction in migraine disability was demonstrated by the mean HIT-6 score being significantly improved from baseline (67.2 ± 0.7) to the end of the nVNS treatment period (64.1 ± 0.7; *P* < 0.001) (Fig. 4). This improvement of 3.1 exceeds the estimated minimally important difference (MID) of 2.3 to 2.7 that has been established as clinically meaningful [21].

**Fig. 5:**
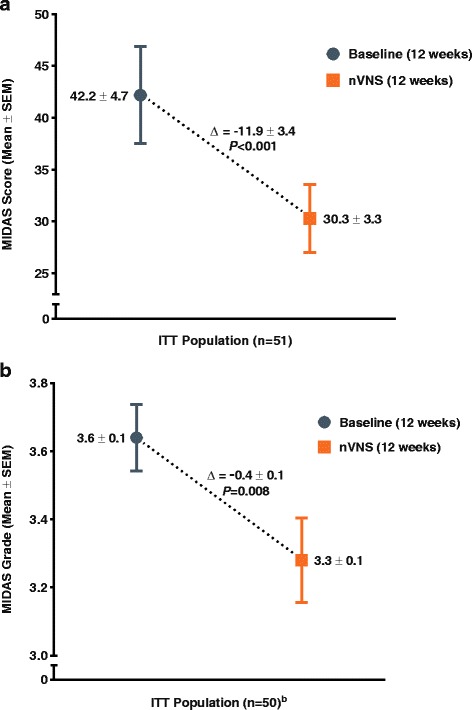
Change in MIDAS Migraine Disability (ITT Population)^a.^ Abbreviations: ITT, intention-to-treat; MIDAS, Migraine Disability Assessment; nVNS, non-invasive vagus nerve stimulation; SEM, standard error of the mean. MIDAS score (**a**) and MIDAS grade (**b**). ^a^Data are mean ± SEM; mean differences between treatment groups may not reflect calculated differences because of rounding; *P* values were from the *t* test; missing data were imputed using the last observation carried forward. ^b^One subject had missing MIDAS grade data for both the baseline and nVNS periods

Significant improvements after nVNS treatment were also observed for MIDAS score (baseline, 42.2 ± 4.7; nVNS, 30.3 ± 3.3; *P* < 0.001) (Fig. 5a) and MIDAS grade (baseline, 3.6 ± 0.1; nVNS, 3.3 ± 0.1; *P* = 0.008) (Fig. 5b).

### Pain intensity

**Fig. 6:**
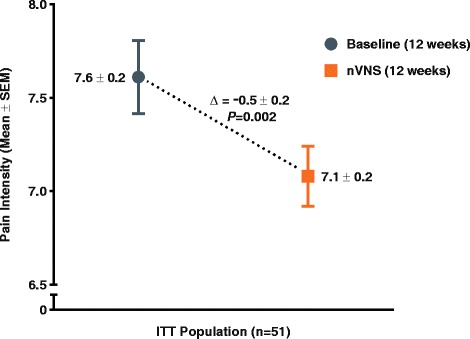
Change in Pain Intensity (ITT Population)^a.^ Abbreviations: ITT, intention-to-treat; nVNS, non-invasive vagus nerve stimulation; SEM, standard error of the mean. ^a^Data are mean ± SEM; mean differences between treatment groups may not reflect calculated differences because of rounding; *P* values are from the *t* test; missing data were imputed using the last observation carried forward

Mean pain intensity demonstrated a clinically modest reduction from baseline (7.6 ± 0.2) to the end of the nVNS treatment period (7.1 ± 0.2; *P* = 0.002) (Fig. 6).

### Allodynia

Allodynia scores during the treated migraine attacks were similar between the two study periods, with a mean difference of 0.2 ± 0.4 between the baseline period (5.4 ± 0.7) and the nVNS treatment period (5.5 ± 0.8; *P* = 0.58).

### Safety and tolerability

There were no safety concerns during the study. The most commonly self-reported AEs were mild or moderate application site reactions (e.g., tingling) and facial/neck twitching. These AEs were transient in nature, coincided with the stimulation period, and resolved during treatment. No serious AEs occurred. Only 1 subject discontinued from the nVNS treatment period because of non–device-related AEs, which included dizziness and anxiety.

## Discussion

To our knowledge, this study is the first to evaluate the effects of a neuromodulation device for the treatment of MM/MRM without aura. Primary end point results demonstrated that prophylactic nVNS administered from −3 days before estimated onset of menstruation through +3 days after the end of menstruation (a total of 10 to 14 days of treatment per month) for 3 cycles provided an average decrease of 2.4 MM/MRM days per month (~33 %), which exceeds the MID of 1 headache day established for this end point [24, 25]. The change in number of headache days from baseline is among the few headache-symptom measures with an established MID, and this MID has been associated with significant effects on health-related quality of life [24, 25]. The assessment of MM/MRM days for the primary end point is clinically relevant to patients and treating physicians, acknowledging that we did not concurrently assess the frequency of individual attacks in this initial study of MM/MRM prophylaxis. The reduction in MM/MRM days was accompanied by significant and clinically meaningful reductions in analgesic use and functional disability. Although this prophylactic study was not intended to evaluate the efficacy of nVNS in the acute treatment of attacks, a modest decrease in pain intensity was observed for the attacks that were not prevented. As several end points in this study represent monthly averages during each 3-month study period, the possibility for improvements in actual monthly MM/MRM outcomes with nVNS treatment over time warrants further evaluation.

Study limitations include the open-label, single-arm study design and the potential for inaccurate subject predictions of menses onset. The open-label study design likely contributed a placebo response to the treatment, as seen in other therapeutic migraine studies [18, 26]. In this study, the actual treatment effect of nVNS cannot be separated from the potential placebo effect because of the lack of a sham control group. On the basis of the ease of use and lack of AE concerns associated with nVNS in the current study, the device appears to have a favourable risk-benefit profile in MM/MRM that is appropriate for further evaluation. As in any MM/MRM study of prophylaxis, imprecise treatment initiation due to inaccurate predictions of the start of menses may have affected the observed results [12] but is likely reflective of real-world use; nVNS has the flexibility to be used outside the perimenstrual period (e.g., before or after menstruation) without increasing the potential for medication-related AEs and complications.

The relative refractoriness of MM/MRM attacks to pharmacologic treatments currently available (but not specifically approved) for the condition [10] may be related to the complexity of the relationship between migraine and oestrogen and the brain’s sensitivity to fluctuations in hormones that cross the blood–brain barrier [27]. Chronic exposure to oestrogen has been suggested to enhance susceptibility to migraine by promoting increased numbers and excitation of glutamatergic neurons [28]. Paradoxically, oestrogen withdrawal is associated with the occurrence of MM/MRM attacks [27]. The efficacy of nVNS in the treatment of migraine may be related to the suppression of excessive extracellular levels of glutamate [29]. In MM/MRM, prophylactic nVNS may exert its anti-glutamatergic mechanistic actions directly via the trigeminal nociceptive pathways of women who are susceptible to oestrogen-related attacks.

The efficacy of prophylactic medications used for MM/MRM has been demonstrated in clinical trials [30, 31] but may be limited by oestrogen level declines and by concerns related to rebound headaches, practicality, and safety. Managing optimal oestrogen levels is challenging for many clinicians, especially in the context of orally administered drugs, because of the complex changes that occur throughout the menstrual cycle [27]. Randomised, double-blind, placebo-controlled MM/MRM studies have suggested that prophylactic triptan and perimenstrual oestrogen therapy may lead to post-treatment or rebound migraine attacks [32–34], possibly reflecting a delayed abrupt decrease in oestrogen levels upon medication discontinuation or a nonhormonal occurrence of end-menstrual migraine (i.e., related to blood loss and transient relative anaemia) [35]. Rebound attacks were not observed after nVNS discontinuation in the current trial or in previous studies of primary headache conditions [16–19], but additional studies are needed to rule out the potential for post-nVNS migraines. Patients with MM/MRM may be reluctant to accept these and other AE-related consequences of preventive triptans given the potentially modest therapeutic gain [12, 15, 32]. The oral anticonvulsant topiramate has been shown to be effective in the prevention of MM attacks but does not appear to reduce attack severity or duration [36]. In addition, topiramate may require several months of daily administration to achieve its potential benefits and may increase the risk for serious adverse events [36, 37]. Non-invasive vagus nerve stimulation is a practical nonpharmacologic option that does not affect oestrogen levels, interact with acute medications, or increase cardiovascular disease risk. Results of the current study suggest that prophylactic nVNS reduces medication use in women with MM/MRM, thereby possibly mitigating the future risk of medication overuse headache and chronic migraine [13, 16, 38].

Non-invasive vagus nerve stimulation may serve as an effective therapeutic option with no safety or tolerability concerns for MM/MRM prevention and may mitigate the risk of medication-related AEs and complications, rebound headaches, and potential drug interactions that are highly relevant among this population. Prophylactic use of nVNS for up to 14 days per month is practical and appears to provide a clinical benefit in MM/MRM while decreasing the need for analgesic medications, consistent with results of previous nVNS studies of other primary headache disorders [16–19].

## Conclusions

This study suggests that mini-prophylaxis with nVNS is an effective treatment that reduces the number of MM/MRM days and acute analgesic use for subjects with MM/MRM without adding any treatment-related safety or tolerability concerns. The current study also expands the body of evidence regarding this condition, for which there are no specifically approved therapies. As the first neuromodulation technique evaluated in patients with MM/MRM, nVNS may offer an important new option for effectively treating the condition while mitigating the monthly perimenstrual AE burden and potential complications of pharmacologic medications. Randomised controlled studies are needed to validate these results.

## Abbreviations

AE: Adverse event
ASC-12: Allodynia symptom checklist
CI: Confidence interval
HIT-6™: 6-item Headache Impact Test
ICHD-III beta: International classification of headache disorders, 3rd edition (beta version)
IRCCS: Istituto di Ricovero e Cura a Carattere Scientifico
ITT: Intent-to-treat
MID: Minimally important difference
MIDAS: Migraine disability assessment
MM: Menstrual migraine
MRM: Menstrually related migraine
NSAID: Non-steroidal anti-inflammatory drug
nVNS: Non-invasive vagus nerve stimulation
SEM: Standard error of the mean
